# Efficacy of Transcranial Direct Current Stimulation (tDCS) on Neuropsychiatric Symptoms in Substance Use Disorder (SUD)—A Review and Insights into Possible Mechanisms of Action

**DOI:** 10.3390/jcm14041337

**Published:** 2025-02-18

**Authors:** James Chmiel, Marta Stępień-Słodkowska, Irena Ramik-Mażewska

**Affiliations:** 1 Institute of Physical Culture Sciences, Faculty of Physical Culture and Health, University of Szczecin, Al. Piastów 40B, Block 6, 71-065 Szczecin, Poland; 2 Doctoral School, University of Szczecin, Mickiewicza 16, 70-384 Szczecin, Poland; 3 Institute of Pedagogy, University of Szczecin, ul. Ogińskiego 16/17, 71-415 Szczecin, Poland

**Keywords:** tDCS, transcranial direct current stimulation, substance use disorder, addiction, dependence, depression, anxiety, noninvasive brain stimulation, neurostimulation, neuromodulation

## Abstract

**Introduction:** Substance use disorder (SUD) is a significant global clinical issue marked by the excessive consumption of alcohol, nicotine, and various psychoactive substances, leading to impaired social, cognitive, and occupational functioning. Individuals with SUD frequently experience depression and anxiety disorders, which exacerbate their prognosis and contribute to substantial health and social burdens. The pathophysiology of SUD and its associated conditions is multifaceted, involving multiple dysfunctions in the brain. This complexity underscores an urgent need for the development of noninvasive treatments that can directly target the brain. One of them is transcranial direct current stimulation (tDCS), an intensively studied technique for safely modulating cortical excitability. The aim of this study is to investigate the effectiveness of tDCS in treating symptoms of depression and anxiety in SUD. **Methods:** With an emphasis on the underlying mechanisms of action, this mechanistic review investigates the effectiveness of tDCS in treating anxiety and depression in SUD patients. Literature searches were conducted using the PubMed/Medline, ResearchGate, Cochrane, and Google Scholar databases. **Results:** The review identified 12 relevant studies. The results showed that left dorsolateral prefrontal cortex (DLPFC) stimulation is an effective treatment option for depression in SUD. In anxiety disorders, left and right DLPFC stimulation is effective, with better results observed with right DLPFC stimulation. However, the included studies differed in their methodology, sample characteristics, and measurement methods, which could have influenced the final results of the analysis. The central focus of this mechanistic review is to discuss the potential mechanisms of action of tDCS in treating depression and anxiety in SUD. These mechanisms include the modulation of brain networks, a reduction in neuroinflammation, an enhancement in neuroplasticity, and an increase in P300 amplitude. We also discuss the limitations of the included studies and propose ways to address them in future research. **Conclusions:** This review provides evidence that tDCS is an effective treatment option for anxiety and depression in SUD. Stimulation of the left DLPFC reduces symptoms of depression, while stimulation of the right DLPFC reduces symptoms of anxiety. However, future research is required to confirm these findings and to deepen our understanding of the mechanisms through which tDCS exerts its effects in this context. Neuroimaging methods (fMRI and EEG) and blood tests could be particularly useful.

## 1. Introduction

Substance use disorder (SUD) represents a significant global clinical challenge marked by the excessive consumption of alcohol, nicotine, and various psychoactive substances, leading to impaired social, cognitive, and occupational functioning. Commonly misused illegal substances include cannabis, sedatives, hypnotics, anxiolytics, inhalants, opioids, hallucinogens, and stimulants. SUD is typically defined by three core features: physical or psychological dependence, intoxication, and abuse [1]. Its prevalence varies across countries and depends on factors such as the type of substances used (most commonly tobacco and alcohol) and demographic or socioeconomic factors. Higher prevalence rates are observed among younger populations and men, although these rates tend to decline with age in both sexes [2]. Additionally, SUD is frequently associated with other neuropsychiatric conditions, such as anxiety and depression, which complicate treatment efforts and exacerbate the overall health burden.

Major depressive disorder (MDD) commonly co-occurs with SUD, posing significant challenges for clinical management and research [3]. Both MDD and SUD independently contribute to substantial global morbidity, and their combination amplifies risks such as suicide, social dysfunction, and resistance to treatment [4]. Approximately one-third of individuals with MDD meet the criteria for SUD [5], with alcohol use disorder (AUD) and illicit drug misuse being particularly prevalent in this population [6,7]. Women with MDD are more likely than men to have a comorbid SUD [5], although men report higher overall levels of substance misuse [8]. In clinical settings, the prevalence of MDD–SUD comorbidity often exceeds that observed in the general population.

The comorbidity of depression and SUD is underpinned by shared neurobiological mechanisms involving critical brain regions, neurotransmission, and molecular pathways [9]. The mesolimbic dopamine circuit—particularly the ventral tegmental area (VTA) and nucleus accumbens (NAc)—is central to both mood regulation and substance reinforcement. In depression, reduced activity in this reward pathway contributes to anhedonia, manifested by diminished responsiveness to pleasurable stimuli [10,11]. In SUD, repeated drug exposure initially triggers excessive dopamine release within these regions, followed by compensatory downregulation, leading to depressive-like states during withdrawal [12].

The prefrontal cortex (PFC), which governs executive functions like decision-making and impulse regulation [13], is also implicated in both disorders. Decreased PFC activity makes it harder to regulate emotions and resist compulsive behaviors. Chronic stress and prolonged substance use further compromise this brain region, perpetuating both mood instability and drug-seeking behaviors [14,15,16].

The dysregulation of multiple neurotransmitter systems forms a central aspect of depression and SUD, contributing to their frequent co-occurrence. Dopamine, serotonin, gamma-aminobutyric acid (GABA), and glutamate are among the key neurotransmitters shaping the clinical trajectory of these conditions. Dopamine is crucial for reward and motivation in both disorders. In MDD, deficient dopamine activity manifests as anhedonia [11,12], while in SUD, substances initially stimulate dopamine release in the mesolimbic pathway, producing euphoric effects that reinforce substance use. Over time, however, receptor downregulation and decreased synthesis dull reward sensitivity, exacerbate depressive symptoms during withdrawal, and reinforce drug cravings, creating a vicious cycle.

Serotonin, closely tied to mood regulation, is also disrupted in both SUD and MDD. In depression, reduced serotonin levels contribute to mood instability and impulsive behaviors [17,18]. Substances such as alcohol, MDMA, and cocaine can temporarily boost serotonin levels by increasing its release or inhibiting its reuptake, leading to short-term mood enhancement. However, chronic use eventually depletes serotonin reserves and impairs receptor functionality, escalating depressive symptoms and emotional instability [19,20,21]. This depletion also increases vulnerability to stress, further entwining SUD and depression.

GABA and glutamate—the main inhibitory and excitatory neurotransmitters, respectively—also exhibit significant dysregulation in SUD and MDD [22]. The long-term use of substances like alcohol or benzodiazepines initially enhances GABA’s inhibitory and calming effect but eventually leads to receptor tolerance and less effective inhibitory control, spurring anxiety and stress during withdrawal [23,24,25,26,27]. Conversely, drugs such as cocaine and methamphetamine disrupt glutamatergic signaling, fueling excitotoxicity and emotional instability [28]. In MDD, excessive glutamate activity has been linked to neurotoxic processes and impaired synaptic plasticity, exacerbating cognitive deficits and emotional dysregulation [29].

The relationship between depression and SUD is inherently bidirectional, with each condition capable of precipitating or exacerbating the other. Depression often drives individuals toward substance use as a form of self-medication. The hallmark symptoms of MDD, such as persistent low mood, anhedonia, and anxiety, can prompt individuals to seek temporary relief through substances like alcohol, opioids, or stimulants. For instance, alcohol is commonly consumed for its sedative effects, providing a short-lived reprieve from emotional distress. However, this relief is often followed by a rebound effect, where the depressive symptoms return with greater intensity, further reinforcing substance use. Similarly, stimulants like cocaine or amphetamines are often used to counteract fatigue or apathy but can lead to mood deterioration during withdrawal phases. Conversely, chronic substance use can induce or worsen depressive symptoms. Prolonged exposure to substances disrupts the brain’s reward pathways and neurotransmitter systems, such as serotonin and dopamine, which are critical for mood regulation. Alcohol, a central nervous system depressant, gradually lowers baseline mood levels, potentially leading to alcohol-induced depressive episodes. Although substances like cocaine and opioids initially elevate mood through heightened dopamine release, they often result in depressive crashes during withdrawal. These crashes are characterized by anhedonia, lethargy, and despair.

Anxiety disorders and SUD frequently co-occur, creating complex challenges for both diagnosis and treatment. Research highlights high rates of co-occurrence, with up to 17.7% of individuals with SUD meeting the criteria for an anxiety disorder, and many individuals with anxiety disorders develop substance-related issues [30]. Specific conditions, such as post-traumatic stress disorder (PTSD), generalized anxiety disorder (GAD), and social anxiety disorder, show particularly strong links with substance use, with PTSD exhibiting some of the highest comorbidity rates [31].

Three primary pathways describe the relationship between anxiety disorders and SUD. The first is the self-medication hypothesis, which suggests that individuals use substances such as alcohol, cannabis, or opioids to alleviate anxiety symptoms. This pathway is commonly observed in conditions like social anxiety and PTSD, where substances may temporarily dampen emotional distress [32]. The second pathway involves substance-induced anxiety, in which certain substances can cause or exacerbate anxiety symptoms, particularly during withdrawal or chronic use. Stimulants, such as cocaine and amphetamines, are particularly implicated in this mechanism [33]. Lastly, the shared vulnerability model proposes that common genetic, neurobiological, or psychosocial factors predispose individuals to both conditions. Early trauma, personality traits like anxiety sensitivity, and disruptions in stress response systems are key shared risk factors [34].

Co-occurring anxiety and SUD are associated with greater symptom severity and functional impairment than either condition alone [35]. This combination also leads to higher rates of relapse and treatment dropout if both conditions are not addressed simultaneously. Treatment is further complicated by pharmacological challenges as medications like benzodiazepines, which are effective for anxiety, pose risks for individuals with SUD [35].

The neurobiological mechanisms underlying the relationship between anxiety and SUD involve several processes, particularly the hypothalamic–pituitary–adrenal (HPA) axis and its interaction with stress and reward systems in the brain. The HPA axis is central to the body’s response to stress and is heavily implicated in substance addiction. Chronic substance use, including alcohol, methamphetamine, and cocaine, dysregulates the HPA axis, altering levels of stress hormones such as cortisol and corticotropin-releasing hormone (CRH). This dysregulation perpetuates a cycle of stress-induced substance craving and use [36].

Stress amplifies dopamine release in reward-related brain regions, such as the nucleus accumbens (NAc) and the ventral tegmental area (VTA). Glucocorticoids released during stress facilitate dopamine synthesis and inhibit its reuptake, reinforcing drug-seeking behavior as a maladaptive coping strategy for stress-induced anxiety [37]. Chronic substance use and stress exposure lead to neuroplastic changes in brain regions involved in reward processing, such as the prefrontal cortex, amygdala, and hippocampus. These changes impair executive control and enhance the sensitivity of the reward system to drugs, further linking stress, anxiety, and addiction vulnerability [38].

SUD is a condition that includes several different types of brain dysfunction, often reflecting concomitant neuropsychiatric conditions. Targeted therapeutic approaches are required to target these brain dysfunctions effectively. One of them is transcranial direct current stimulation (tDCS), a noninvasive brain stimulation technique extensively researched for more than 20 years, which has demonstrated its ability to treat a variety of neurological and psychiatric conditions.

tDCS delivers a low-intensity direct current (usually 0.5–2 mA) to the brain through at least two scalp electrodes—an anode and a cathode—often composed of saline-soaked sponges connected to a battery-powered device [39]. A portion of this current penetrates the skull, modulating neuronal resting membrane potentials [40,41]. Anodal stimulation typically depolarizes neurons, increasing their excitability, while cathodal stimulation hyperpolarizes neurons, reducing their likelihood of firing [42]. The precise effects depend on the orientation of neuronal elements in the targeted cortical region. Although tDCS is inherently non-focal due to the flow of current between electrodes, adjusting the size of the electrode can help localize stimulation to smaller areas [43].

Standard protocols involve sessions of 5 to 30 min, with 20 min being the most common, and current intensities ranging from 0.5 to 2 mA. Even a single session of up to 15 min can alter cortical excitability for roughly 90 min, and when stimulation exceeds 10 min at 1–2 mA, changes often persist for at least an hour [44]. Repeated application can further amplify and prolong these effects. Mechanistically, tDCS engages calcium-dependent synaptic plasticity processes in glutamatergic neurons via N-methyl-D-aspartate (NMDA) receptors, facilitating long-term potentiation (LTP) or long-term depression (LTD), depending on the concentration of intracellular calcium [45]. Even brief stimulation of about three minutes can induce effects lasting beyond the stimulation period, and administration longer than 10 min at 1–2 mA stabilizes these synaptic modifications for at least an hour [40]. Both anodal and cathodal tDCS can also diminish gamma-aminobutyric acid (GABA) levels, rebalancing the cortical excitation–inhibition interplay by influencing neurotransmitters such as glutamate and modulating brain-derived neurotrophic factor (BDNF) [46,47,48,49]. Notably, these changes are not confined to the stimulated region but can extend to functionally connected brain networks [50]. Given its ability to modulate neuroplasticity, tDCS has shown promise in multiple clinical applications, with minimal side effects, mainly mild itching or tingling on the scalp [51].

Numerous reviews have evaluated the efficacy of tDCS in treating SUD [52,53,54,55,56,57,58,59,60,61,62,63,64,65,66,67,68]. Most studies have shown the efficacy of tDCS in reducing cravings, decreasing substance use frequency, and improving cognitive function. However, its potential in improving neuropsychiatric disorders in individuals with SUD has not been studied. This review’s objective is to close this gap by examining how tDCS affects SUD patients’ anxiety and depressive symptoms and whether tDCS is effective in treating these symptoms. In particular, the mechanistic approach used in this research aims to comprehend how tDCS influences neuropsychiatric symptoms, providing insights into the physiological and molecular processes underlying its therapeutic outcomes.

## 2. Methods

The purpose of this mechanistic review is to systematically assess the efficacy of tDCS in alleviating neuropsychiatric symptoms, particularly anxiety and depression, in SUD patients. To ensure the validity and relevance of the included evidence, strict selection criteria and a comprehensive literature search were applied. Following established guidelines for systematic reviews and evidence synthesis (PRISMA) [69], the methodology concentrated on finding case studies and clinical trials that evaluated the therapeutic effect of tDCS on neuropsychiatric dysfunctions associated with SUD. It is crucial to note that this review adopts a mechanistic rather than a fully systematic approach and does not adhere to every aspect of the PRISMA methodology typically used in systematic reviews.

### 2.1. Data Sources and Search Strategy

To compile this evaluation, J.C., M.S.-S. and I.R.-M. conducted an independent standards-compliant Internet search using a combination of targeted keywords. These included “tDCS” or “transcranial direct current stimulation” and “alcohol”, “drinking”, “alcohol”, “craving”, “substance”, “substance use disorder”, “SUD”, “nicotine”, “cigarettes”, “tobacco”, “smoking”, “methamphetamine”, “meth”, “cocaine”, “crack”, “heroin”, “methadone”, “cannabis”, and “marijuana”. A comprehensive search was conducted in December 2024 across multiple databases, including PubMed/Medline, Research Gate, Google Scholar, and Cochrane, focusing on publications published between January 2008 and December 2024.

### 2.2. Study Selection Criteria

To qualify for inclusion, studies had to be clinical trials or case studies published in English between 2008 and 2024. They had to examine the impact of tDCS on anxiety or depression in SUD patients, either as a primary or secondary outcome. Review papers and non-English-language publications were excluded from consideration.

### 2.3. Screening Process

A multi-step screening process was employed to ensure the inclusion of relevant studies and the exclusion of those that did not meet the predefined criteria. In the first screening step, abstracts and titles were carefully examined by three independent reviewers: J.C., M.S.-S, and I.R.-M.

#### 2.3.1. Title and Abstract Screening

During the initial screening, each reviewer evaluated the abstracts and titles of the independently accessible records to determine whether they met the inclusion criteria. The primary focus was on studies exploring the effect of tDCS on anxiety and depression in SUD patients.

#### 2.3.2. Full-Text Assessment

Following the initial screening, the selected papers were subjected to a comprehensive full-text review. The reviewers thoroughly analyzed each publication to confirm eligibility, ensuring the studies were clinical trials or case studies published in English between January 2008 and December 2024.

## 3. Results

**Figure 1:**
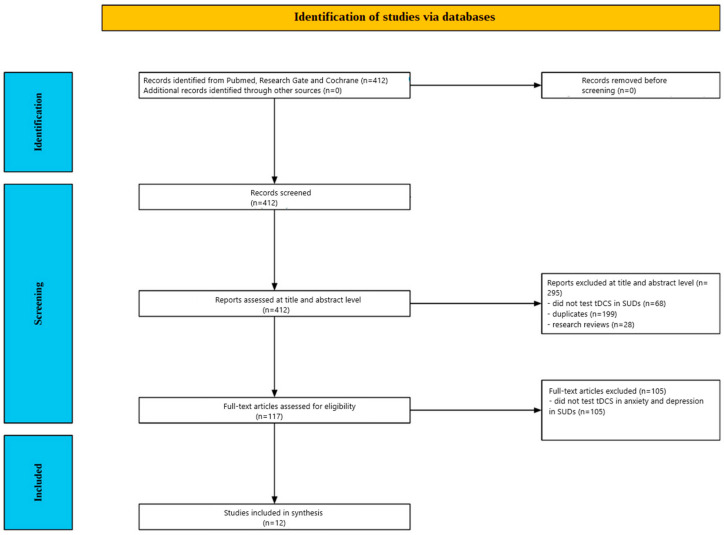
Flow chart depicting the different phases of the systematic review.

The screening procedure is summarized in Figure 1. Of the 412 studies, initially identified through database searches, 295 publications were excluded after title and abstract review. The reasons for exclusion included 68 studies that did not evaluate tDCS in SUD, 199 duplicates, and 28 review articles. The remaining 117 papers underwent a thorough full-text assessment, during which 105 studies were subsequently disqualified for failing to examine the impact of tDCS on anxiety or depression in SUD patients. Ultimately, twelve (12) articles met the inclusion criteria and were included in this review.

The twelve studies [70,71,72,73,74,75,76,77,78,79,80,81], published between 2013 and 2024, utilized six psychometric tools to evaluate neuropsychiatric symptoms: Hamilton Anxiety Rating Scale (HAM-A), Beck Anxiety Inventory (BAI), Hamilton Depression Rating Scale (HAM-D), Beck Depression Inventory-II (BDI-II), Hamilton Depression Rating Scale (HDRS), and Depression, Anxiety, and Stress Scale-21 (DASS-21).

The HAM-A is a clinician-administered scale featuring 14 items scored on a five-point scale. It encompasses both somatic and psychic symptoms of anxiety, with total scores spanning from 0 to 56 to quantify severity. Values under 15 typically signify mild anxiety, 15–23 suggest moderate anxiety, and 24 or above indicate severe anxiety [82].

The BAI is a self-report tool comprising 21 items that rate anxiety symptoms from 0 (not at all) to 3 (severely). It assists clinicians and researchers in categorizing the intensity of anxiety presentations. The BAI produces total scores ranging from 0 to 63 and classifies anxiety as minimal (0–9), mild–moderate (10–18), moderate–severe (19–29), or severe (30+) [83].

The HAM-D/HDRS is a widely recognized clinician-rated scale designed with 17 or more items to capture varied aspects of depression, including mood, guilt, and psychomotor changes. It generates an overall score reflecting the severity of depressive symptoms. It can produce scores up to 52 depending on the version, distinguishing between minimal, mild, moderate, and severe depression. The outcomes are commonly interpreted as 0–7 (normal), 8–16 (mild), 17–23 (moderate), and 24 or more (severe) [84].

The 21-item Beck Depression Inventory-II (BDI-II) uses a 0–3 scale for each item, resulting in total scores of 0–63, with cutoffs for minimal (0–13), mild (14–19), moderate (20–28), and severe (29+) [85].

A shortened version of the original 42-item DASS is the DASS-21, which includes three subscales for depression, anxiety, and stress, with seven items each, scored on a scale of 0 (did not apply at all) to 3 (applied very much). It offers a structured means to evaluate and distinguish between these interrelated emotional states. Although each subscale has its own cutoff ranges, a typical classification for the depression subscale is 0–9 (normal), 10–13 (mild), 14–20 (moderate), 21–27 (severe), and 28+ (extremely severe) [86].

### 3.1. Summary of Included Studies

The included studies are summarized in Table 1. The primary objective of study [70] was to evaluate the effects of tDCS on relapse rates, craving, depressive symptoms, executive functions, and brain activity as measured by event-related potentials (ERPs). Thirteen alcohol-dependent subjects were randomized into two groups: an active tDCS group (*n =* 6) and a sham tDCS group (*n =* 7). Participants received weekly tDCS or sham stimulation sessions for five consecutive weeks. Stimulation parameters included a 2 mA intensity for 20 min, with the anode placed over F3 (left DLPFC) and the cathode over the contralateral supradeltoid area. Depressive and anxiety symptoms were evaluated using the Hamilton Depression Rating Scale (HAM-D) and the Hamilton Anxiety Rating Scale (HAM-A). While the median score of 6 in the HAM-D suggests that most participants did not have significant depressive symptoms at baseline, the wide range (up to 31) indicates that a subset of participants may have had mild to severe depression. For anxiety, the median score of 4 in the HAM-A suggests that most participants did not have significant anxiety at baseline, but the upper range (24) indicates that some participants experienced moderate to severe anxiety.

Study [71] evaluated the effects of tDCS on relapse rates, craving, cognitive function, depression, anxiety, and quality of life in individuals with severe alcohol dependence. The trial involved 33 participants who were randomly assigned to either a real tDCS group (*n =* 16) or a sham tDCS group (*n =* 17). The tDCS protocol involved bilateral stimulation with the cathode over the left DLPFC and the anode over the right DLPFC. Participants received 2 mA stimulation for 13 min, twice daily, for five consecutive days, with a 20 min interval between sessions. The HAM-D was used to measure depression, and participants in the active tDCS group had a mean score of 8.6 ± 8.9 (mild depression). Anxiety, measured using the HAM-A, showed baseline scores of 11.0 ± 9.0 (mild anxiety).

Study [72] investigated the efficacy of anodal tDCS over the right DLPFC in reducing craving, as well as its effects on depressive symptoms, anxiety, impulsiveness, and other psychiatric outcomes in individuals with SUD. This randomized double-blind sham-controlled trial included 34 treatment-seeking subjects diagnosed with SUD or gambling disorder. Participants were randomized into two groups: 18 subjects received active tDCS while 16 received sham stimulation. The tDCS protocol involved placing the cathode over the left DLPFC (F3) and the anode over the right DLPFC (F4), delivering a current of 1.5 mA for 20 min per session over five consecutive days. Depressive symptoms were measured by the HAM-D, with baseline scores of 14.8 ± 7.8, indicating mild depression. Anxiety was assessed using the HAM-A, with baseline scores of 13.3 ± 8.6, reflecting mild anxiety.

Paper [73] explored the efficacy of combining tDCS over the DLPFC with alcohol cue inhibitory control training (AICT) to reduce early alcohol relapse in individuals with severe alcohol use disorder (AUD). This randomized placebo-controlled clinical trial included 125 AUD patients. Patients were divided into one of four experimental conditions in a 2 × 2 factorial design: real tDCS with alcohol cue inhibitory control training (AICT) (*n =* 34), real tDCS with neutral inhibitory control training (NICT) (*n =* 28), sham tDCS with AICT (*n =* 27), and sham tDCS with NICT (*n =* 34). The tDCS protocol involved 20 min sessions of 2 mA intensity applied over five consecutive days, with the anode placed over the right DLPFC (F4) and the cathode over the left DLPFC (F3). During stimulation, participants simultaneously completed inhibitory control training. AICT required responses to be inhibited for alcohol-related cues in a Go/No-Go task, while NICT involved neutral images unrelated to alcohol. The study assessed depression using the Beck Depression Inventory-II (BDI-II). At baseline, participants in all groups exhibited mild-to-moderate depressive symptoms, with mean BDI-II scores as follows: 19.92 in the real tDCS + AICT group (borderline moderate depression), 21.82 in the real tDCS + NICT group (moderate depression), 20.52 in the sham tDCS + AICT group (moderate depression), and 19.26 in the sham tDCS + NICT group (borderline moderate depression).

Study [74] investigated the effects of tDCS applied to the DLPFC on cannabis craving, depression, and working memory in individuals diagnosed with cannabis use disorder. The randomized double-blind sham-controlled trial included 50 male participants who were divided into two equal groups: real tDCS (*n =* 25) and sham tDCS (*n =* 25). The intervention consisted of 20 tDCS sessions delivered twice per week for 20 min at 2 mA intensity. Electrodes were placed at F3 and F4 over the DLPFC. Depression was measured using the Hamilton Depression Rating Scale (outcomes before tDCS: 23.04 ± 4.44, moderate–severe depression).

Study [75] investigated the impact of tDCS on relapse rates, depression, anxiety, and stress in opioid-dependent individuals undergoing methadone maintenance treatment (MMT). This randomized double-blind sham-controlled pilot study included 27 male participants who were divided into an intervention group (*n =* 14) and a sham group (*n =* 13). The intervention group underwent seven sessions of tDCS (20 min each) over two consecutive weeks, with the anode placed on the right DLPFC at F4 and the cathode on the left DLPFC (F3), delivering a 2 mA current. Depression and anxiety were measured using the Depression, Anxiety, and Stress Scale-21 (DASS-21). For depression, the baseline mean score in the intervention group (real tDCS) was 30.71 ± 7.75, reflecting extremely severe depressive symptoms, while in the sham group, the mean score was 27.85 ± 8.46, indicating severe depression. There was no significant difference in baseline scores between the groups. For anxiety, baseline mean scores were 27.00 ± 8.25 in the intervention group and 25.38 ± 9.36 in the sham group, both indicating severe anxiety, with no statistically significant differences between groups.

Study [76] investigated the efficacy of tDCS applied to the DLPFC in combination with methadone maintenance treatment (MMT) in reducing opium craving, depression, and anxiety in patients with opium use disorder. Sixty participants were randomly assigned to one of three groups: (1) active tDCS + MMT (*n =* 20), (2) sham tDCS + MMT (*n =* 20), and (3) MMT-only (*n =* 20). The active tDCS protocol involved placing the anode on the right DLPFC (F4) and the cathode on the left DLPFC (F3), with a current of 2 mA for 20 min per session, conducted once daily for 10 consecutive days. Depression was measured using the Beck Depression Inventory-II (BDI-II), and anxiety was assessed with the Beck Anxiety Inventory (BAI). Baseline mean depression scores were 17.55 ± 6.45 in the active tDCS group (mild depression), 18.70 ± 6.48 in the sham tDCS group, and 17.40 ± 7.07 in the MMT-only group, showing no significant differences between the groups. For anxiety, the baseline scores were 12.10 ± 4.47 in the active tDCS group (mild anxiety), 14.20 ± 4.76 in the sham group, and 14.85 ± 4.99 in the MMT-only group, with no significant differences.

Experiment [77] assessed the effects of tDCS applied to the DLPFC on brain-derived neurotrophic factor (BDNF), depression, anxiety, stress, and craving in opioid-addicted patients undergoing methadone maintenance treatment. Thirty participants were randomly assigned to three groups of ten each: Group A (*n =* 10, anodal left DLPFC/cathodal right DLPFC stimulation), Group B (*n =* 10, anodal right DLPFC/cathodal left DLPFC stimulation), and Group C (*n =* 10, sham tDCS). The intervention consisted of 10 sessions of tDCS lasting 20 min each. No current intensity specified). For depression and anxiety, measured using the Depression, Anxiety, and Stress Scale-21 (DASS-21), the baseline scores for depression were 28.20 ± 8.66 in Group A (extremely severe depression), 29.45 ± 6.99 in Group B (extremely severe depression), and 26.20 ± 7.51 in Group C (severe depression), and baseline scores for anxiety were 24.60 ± 7.77 in Group A (severe anxiety), 24.73 ± 8.86 in Group B (severe anxiety), and 24.00 ± 7.95 in Group C (severe anxiety).

Study [78] investigated the effects of bilateral tDCS on craving, depression, anxiety, and quality of life in individuals with crack-cocaine dependence. Conducted as a double-blind, randomized, and placebo-controlled trial, the study included 36 male participants assigned to either an active tDCS group (*n =* 17) or a sham tDCS group (*n =* 19). Participants underwent five sessions of 20 min tDCS every other day. The intervention used a 2 mA current for 20 min, with the anode placed over the right DLPFC (F4) and the cathode placed over the left DLPFC (F3). Depression was measured using the HAM-D, and anxiety was assessed using the HAM-A. For depression, the mean baseline HAM-D scores were 5.0 in the active tDCS group (no depression) and 4.3 in the sham group, with no statistically significant differences. For anxiety, baseline HAM-A scores were 7.6 in the active tDCS group (normal–mild anxiety) and 6.0 in the sham group, with no significant differences between the groups at pre-treatment.

Study [79] aimed to investigate the effects of tDCS on craving, depression, anxiety, and quality of life in individuals with cocaine use disorder (CUD). Conducted as a double-blind, randomized, and sham-controlled trial, the study recruited 17 participants diagnosed with CUD, who received either real tDCS (*n =* 8) or sham tDCS (*n =* 9). The intervention included 15 sessions of right-anodal/left-cathodal tDCS targeting the DLPFC over a 5-week period. Each session involved the application of 2 mA current for 20 min. For measuring depression, it used the HAM-D, and for anxiety, it used the HAM-A. At baseline, the mean HAM-D scores were 5.6 ± 3.0 (no depression) in the real tDCS group and 4.3 ± 3.1 in the sham group, with no significant differences between the groups. For anxiety, the baseline HAM-A scores were 7.6 ± 5.8 in the real tDCS group (normal–mild anxiety) and 6.0 ± 4.3 in the sham group, again with no significant baseline differences.

Study [80] investigated the efficacy of tDCS in reducing crack craving and its impact on anxiety and cognitive performance. This open-label trial involved 11 male patients diagnosed with crack/cocaine addiction. Participants received 10 consecutive sessions of tDCS over 10 days, with the anode positioned over the left DLPFC and the cathode placed contralaterally. Stimulation was delivered at 2.0 mA for 20 min per session, with electrode placement guided by the Beam F3 System for precise localization of the DLPFC. Anxiety symptoms were assessed using the Beck Anxiety Inventory (BAI). At baseline, the mean BAI score was 8.8, indicating minimal to mild anxiety levels.

The aim of study [81] was to examine the combined effects of tDCS and Mindfulness-Based Substance Abuse Treatment (MBSAT) on negative emotions (depression, anxiety, and stress) and craving in adolescents with methamphetamine dependence. The study included 80 adolescents randomly assigned to one of four intervention groups: (1) the tDCS group, which received tDCS only (*n =* 20), (2) the MBSAT group, which underwent mindfulness therapy alone (*n =* 20), (3) the combined group (tDCS + MBSAT, *n =* 20), which received both tDCS and mindfulness therapy, and (4) the sham group, which received placebo (sham) tDCS (*n =* 20). The intervention consisted of 12 sessions over six weeks, each lasting approximately 50 min. tDCS was applied at 1.5 mA for 20 min over the left DLPFC, although the placement of the cathodal electrode was not specified. The DASS-21 was used to measure anxiety and depression. Although no numerical information was provided for these measures, graphical estimates suggest that both depression and anxiety scores were approximately 13, reflecting mild anxiety and depression.

### 3.2. Impact on Depression

Study [70] demonstrated that after five weekly sessions of tDCS, the active tDCS group showed a significantly greater reduction in depression symptoms compared to the sham group. The median change in HAM-D scores was −6 (range: −20 to −3) in the active tDCS group, whereas it was only −1 (range: −3 to 14) in the sham group, with a statistically significant difference of *p* = 0.005.

The results from [71] reported reductions in depressive symptoms in both groups following treatment, with a score of 6.8 ± 1.7 (no depression) in the tDCS group (20.93% reduction) and a score of 5.7 ± 4.8 (39.36% reduction) in the sham group. However, the difference between groups was not significant. Within-group comparisons revealed that the sham group experienced a significant reduction in HAM-D scores (*p* = 0.01), while the improvement in the real tDCS group was not statistically significant (*p* = 0.22).

Study [72] found significant reductions in HAM-D scores over time in both groups (*p* < 0.001). The active tDCS group showed a 48.65% reduction from 14.8 ± 7.8 to 7.6 ± 5.1 (no or mild depression), whereas the sham group exhibited a 26.61% reduction (from 12.4 ± 8.2 to 9.1 ± 7.4). Although the active group demonstrated a stronger reduction, the difference between groups did not reach statistical significance (*p* = 0.063).

In study [73], reductions in BDI-II scores showed that the real tDCS + AICT group decreased from 19.92 to 14.72 (mild depression, 26.10% reduction), the real tDCS + NICT group decreased from 21.82 to 17.5 (19.80% reduction), the sham tDCS + AICT group decreased from 20.52 to 15.13 (26.27% reduction), and the sham tDCS + NICT group decreased from 19.26 to 16.04 (16.72% reduction). These results demonstrate that all groups experienced reductions in depressive symptoms, but there were no significant differences between real and sham tDCS or AICT and NICT.

In study [74], the real tDCS group showed significant improvement, with HDRS scores decreasing from 23.04 ± 4.44 at baseline to 15.40 ± 4.49 post-treatment (mild depression), representing a 33.16% reduction (*p* < 0.0001). In the sham group, depression scores showed minimal change, decreasing from 20.32 ± 4.13 to 19.76 ± 3.80, a 2.76% reduction that was not statistically significant (*p* = 0.110).

In study [75], depression scores decreased significantly in the intervention group to 17.57 ± 4.45 after the seventh tDCS session (moderate depression, 42.79% reduction), whereas the sham group showed a smaller reduction, with scores decreasing to 25.85 ± 7.09 (7.18% reduction). Statistical analysis confirmed that the reduction in depression scores in the intervention group was statistically significant compared to the sham group (*p* < 0.001).

In research [76], after 10 days of treatment, depression scores in the active tDCS group significantly decreased to 14.05 ± 6.18 (19.94% reduction), while the sham tDCS group showed only a minor reduction to 18.30 ± 6.89 (2.14% reduction), and the MMT-only group exhibited a slight change to 18.10 ± 6.75. Statistical analysis revealed a significant group × time interaction effect (*p* = 0.009), indicating that the active tDCS group experienced a significantly greater reduction in depressive symptoms compared to the other two groups. Paired-sample t-tests further confirmed that the decrease in depression scores was statistically significant within the active tDCS group (*p* = 0.01), whereas no statistically significant changes were observed in the sham tDCS (*p* = 0.25) and MMT-only groups (*p* = 0.40).

In paper [77], depression scores were reduced significantly in Group A to 14.40 (moderate depression) and in Group B to 17.09 (moderate depression), while the sham group (Group C) showed a slight increase to 26.80. Group A demonstrated the most substantial improvement, with a 48.93% reduction in depression scores, followed by Group B, with a 41.96% reduction, whereas the sham group showed a negligible increase of 2.29%. Statistical analysis confirmed that the reduction in depression scores for Group A was statistically significant compared to Group C (*p* = 0.023), while Group B’s improvement, although notable, did not reach the same level of statistical significance compared to the sham group.

The results from [78] showed that depression scores in the active tDCS group decreased to 3.2 (36.00% reduction), showing a statistically significant improvement within the group (*p* = 0.04). In the sham group, scores showed only a slight decrease to 3.5, and between-group analysis did not reveal a statistically significant difference (*p* = 0.45).

In study [79], depression scores in the real tDCS group decreased to 3.3 (41.07% reduction), whereas the sham group showed a change to 4.8 (11.63% increase). Within-group analysis revealed that the reduction in depression scores in the real tDCS group was statistically significant (*p* = 0.04), while the between-group comparison did not reach significance (*p* = 0.45).

Study [81] showed significant reductions in depression scores across all intervention groups, with the combined tDCS + MBSAT group demonstrating the greatest improvement compared to the other groups. Both the tDCS and MBSAT groups also experienced significant reductions in depression scores compared to the sham group, but the combined approach produced the most pronounced and sustained effects. The mixed-model ANOVA analysis revealed a significant main effect of group and time (*p* < 0.001), with post-hoc comparisons indicating that the combined group outperformed both the individual tDCS and MBSAT groups, as well as the sham group.

### 3.3. Impact on Anxiety

In study [70], the active tDCS group demonstrated a greater numerical reduction in anxiety symptoms compared to the sham group, but the difference did not reach statistical significance. The median change in HAM-A scores was −5.5 (range: −24 to 2) in the active tDCS group and −2 (range: −3 to 14) in the sham group (*p* = 0.169).

In [71], both groups showed improvements (real tDCS = 7.1 ± 7.3, no anxiety, 35.45% reduction), but the difference between groups was again non-significant. Within-group analysis showed a borderline significant improvement in the sham group (*p* = 0.05) and a trend toward improvement in the real tDCS group (*p* = 0.06).

In study [72], anxiety symptoms measured with the HAM-A improved significantly over time (*p* < 0.001), with scores in the active tDCS group decreasing from 13.3 ± 8.6 to 7.1 ± 5.8 (no anxiety, 46.62% reduction) and scores in the sham group decreasing from 9.9 ± 7.0 to 7.8 ± 6.2 (21.21% reduction). A trend favoring the active group was observed but was not statistically significant (*p* = 0.053).

In study [75], anxiety scores in the intervention group decreased significantly to 16.86 ± 4.20 (moderate anxiety, 37.56% reduction), while the sham group showed only a slight reduction to 23.23 ± 7.68, 8.47% reduction). The between-group difference in anxiety reduction was also statistically significant (*p* = 0.01), demonstrating that real tDCS significantly reduced anxiety symptoms compared to sham stimulation.

In study [76], anxiety scores in the active tDCS group decreased significantly to 9.7 ± 3.58 (19.83% reduction), while the sham group showed a slight increase to 14.54 ± 4.64 (2.39% change), and the MMT-only group showed a marginal decrease to 14.05 ± 4.67 (5.39% reduction). Although the group × time interaction effect did not reach statistical significance (*p* = 0.10), there was a significant main effect for time (*p* = 0.04), indicating overall improvement. Paired-sample t-tests revealed that only the active tDCS group experienced a significant reduction in anxiety scores (*p* = 0.02), while the sham tDCS group (*p* = 0.32) and MMT-only group (*p* = 0.24) showed no meaningful changes.

The results from [77] showed that after 10 sessions, anxiety scores decreased significantly to 9.60 in Group A (normal–mild anxiety) and to 11.82 in Group B (mild anxiety), while Group C showed only a slight reduction to 23.20 (severe anxiety). Group A achieved a 60.97% reduction in anxiety scores, and Group B showed a 52.20% reduction, whereas the sham group exhibited only a minor reduction of 3.33%. Statistical analysis revealed significant reductions in anxiety for Group A compared to Group C (*p* = 0.001) as well as Group B compared to Group C (*p* = 0.006).

In study [78], anxiety scores in the active tDCS group decreased to 6.4 (normal anxiety, 15.79% reduction), while in the sham group, scores increased to 8.7 ± 6.5 (45.00% increase). Although the reduction in the active tDCS group was not statistically significant within the group (*p* = 0.30), the sham group demonstrated a near-significant increase in anxiety levels (*p* = 0.053). Importantly, the between-group analysis showed a statistically significant difference (*p* = 0.03), indicating that active tDCS effectively reduced anxiety symptoms compared to sham stimulation.

In study [79], anxiety scores in the real tDCS group decreased to 5.3 (30.26% reduction), while scores in the sham group increased to 9.3 (55.00% increase). Although the within-group reduction in anxiety in the real tDCS group was not statistically significant (*p* = 0.30), the sham group showed a near-significant increase in anxiety levels (*p* = 0.053). Importantly, the between-group comparison showed a statistically significant group × time interaction (*p* = 0.03), indicating that active tDCS was effective in reducing anxiety compared to sham stimulation.

In study [80], there was no significant reduction in BAI scores (*p* = 0.624) (no numerical data).

Study [81] showed significant reductions in anxiety scores were observed across the intervention groups. The combined tDCS + MBSAT group achieved the greatest reduction in anxiety scores compared to the other groups, including the sham group. The MBSAT group and tDCS group independently showed significant improvements, although the effects were not as substantial as in the combined intervention group. The statistical analysis confirmed a significant main effect of group and time (*p* < 0.001), with the combined group showing a clear advantage over the sham and individual treatment groups.

## 4. Discussion

This mechanistic review examined the efficacy of tDCS in treating depression and anxiety in individuals with substance use disorders, analyzing 12 studies that met the inclusion criteria. Of these, 11 focused on depression, while 10 examined anxiety.

Regarding depression, the reviewed studies suggest that tDCS targeting the DLPFC shows promise as an adjunctive intervention for reducing depressive symptoms among individuals with substance dependence. Across diverse populations—including those with alcohol, opioids, cannabis, and cocaine use disorders—tDCS was generally associated with reductions in depression scores, albeit the statistical significance and clinical impact varied.

Several patterns and correlations emerged from the analysis. First, the baseline severity of depression varied widely across studies, ranging from mild to extremely severe, reflecting the heterogeneity of comorbid affective states among substance-abusing populations. Studies involving individuals with more severe baseline depression (e.g., [74,75,77]) reported more pronounced improvements following active tDCS. For instance, in study [74], patients with severe depression experienced substantial reductions in HAM-D scores following active tDCS, while the sham group did not. Similarly, in studies [75,77], participants with severe to extremely severe depression achieved significant benefits from active tDCS, often outperforming sham and other control conditions, including methadone maintenance alone.

Second, the placement of electrodes and the polarity of stimulation (anodal over the right DLPFC and cathodal over the left, or vice versa) did not yield consistent superiority of any specific configuration. Studies using right-anodal stimulation (e.g., [75,76,77]) reported notable improvements, but left-anodal configurations and bilateral montages also demonstrated benefits. This inconsistency may reflect differences in sample characteristics, the neurobiology of different substance use disorders, differences in study design (such as session frequency and total number of sessions), and the role of the DLPFC in regulating affect and executive function. This shows the need for a more systematic exploration of electrode placement and polarity as moderators of tDCS outcomes.

Third, while several studies demonstrated significant within-group reductions in depressive symptoms for participants receiving active tDCS, between-group differences (active vs. sham) often approached but did not consistently reach statistical significance. For example, studies [71,72,73,78] and [79] showed improvements in both the real and sham conditions, suggesting a potential placebo effect or non-specific benefits of study participation, psychosocial support, or concurrent treatments. This aligns with the broader literature, where expectations [87,88] and concurrent rehabilitation therapies can influence subjective mood improvements [89]. More controlled investigations are needed to isolate tDCS-specific effects from these confounding factors.

Fourth, studies combining tDCS with other interventions, such as alcohol cue inhibitory control training [73] or Mindfulness-Based Substance Abuse Treatment (MBSAT) [81], yielded mixed results. While study [73] did not find a significant additive effect of tDCS over AICT compared to sham, study [81] identified a synergistic benefit when tDCS was paired with mindfulness training. This suggests that tDCS may be most effective when integrated into a multi-modal treatment framework that addresses both neurobiological and psychological components of addiction and mood dysregulation. tDCS has been shown to induce neuroplastic changes [90] that may induce beneficial effects in the treatment of substance addiction. Similarly, mindfulness meditation has been shown to be effective in treating substance addiction, and its healing effects also involve inducing a number of neuroplastic mechanisms [91]. Meditation, including mindfulness, in combination with tDCS is gaining popularity, and this approach has been shown to be effective in alleviating a range of conditions [92]. This combined approach may enhance neuroplastic changes induced by tDCS and strengthen newly learned coping skills acquired through behavioral interventions.

Fifth, the type of substance use disorder may influence responsiveness to tDCS. While positive trends were observed with regard to depression across all substance groups studied (alcohol, opioids, cannabis, and stimulants such as cocaine and crack), direct comparisons between substance types remain limited. Understanding how different substance use disorders respond to tDCS could guide individualized treatment approaches and optimize stimulation parameters based on the neurocircuitry affected by each substance.

Lastly, the lack of uniformity in study designs, stimulation parameters, sample sizes, and clinical assessments complicates definitive conclusions. Differences in session frequency, treatment duration, electrode placement, current intensity, and the inclusion of ancillary therapies highlight the need for standardization. Future research should aim to employ larger sample sizes, uniform methodologies, and more rigorous sham control conditions.

Based on the evidence presented, anodal stimulation over the left DLPFC paired with cathodal stimulation over the right DLPFC may be the most effective for improving depressive symptoms in substance-addicted individuals. Across multiple studies, including those comparing different electrode placements and configurations, the most substantial and consistent reductions in depression scores have been observed when the left DLPFC receives anodal stimulation. For instance, study [70] demonstrated a significant decrease in depression scores with the anode over the left DLPFC, while study [74] achieved notable improvements using a bilateral DLPFC montage likely involving left-sided anodal placement. Study [77] further confirmed this pattern, showing that anodal stimulation over the left DLPFC, as opposed to the right, resulted in greater decreases in depression scores. Additionally, study [81] reported pronounced reductions in depression when tDCS at the left DLPFC was combined with mindfulness-based treatment, indicating that this approach enhances therapeutic outcomes. Taken together, these findings suggest that anodal stimulation of the left DLPFC, coupled with cathodal placement over the right DLPFC or an equivalent contralateral site, represents a highly promising tDCS protocol for reducing depressive symptoms in individuals with substance use disorders.

In sum, the reviewed evidence suggests that tDCS targeting the DLPFC can attenuate depressive symptoms in individuals with substance use disorders, with certain configurations and concurrent interventions proving more effective than others. Although improvements were frequently observed in active tDCS groups, the magnitude and specificity of these effects varied. Further well-controlled and large-scale studies are needed to refine the protocols, determine optimal electrode placements and stimulation intensities, and identify patient characteristics most predictive of a robust tDCS-mediated antidepressant response. Ultimately, tDCS may serve as a valuable adjunct to standard treatments, addressing the challenging interplay between mood dysregulation and substance abuse.

The reviewed studies collectively indicate that tDCS applied to the DLPFC has the potential to reduce anxiety symptoms in individuals with various substance use disorders. Although the magnitude and statistical significance of these improvements varied across trials, several noteworthy patterns and correlations emerged.

A consistent finding was that active tDCS tended to yield greater reductions in anxiety compared to sham conditions. For example, studies [75,77,78,79] reported more pronounced decreases in anxiety scores following active tDCS, often with statistically significant between-group differences favoring real stimulation. In study [75], where participants presented with severe baseline anxiety, the active tDCS group significantly outperformed the sham group. Similarly, studies [77,78] found substantial and significant anxiety reductions in active tDCS groups compared to the sham group, demonstrating that active stimulation can lead to clinically meaningful improvements even within short treatment windows. However, not all studies showed robust or statistically significant differences. In studies [70,71,72,76], while numerical improvements in anxiety were observed for active tDCS recipients, these changes often approached but did not consistently reach conventional significance thresholds. Several factors may explain this variability. Differences in sample sizes and statistical power likely contributed to the mixed findings as some studies recruited relatively small cohorts. In addition, baseline anxiety severity varied widely. Some studies involved participants with mild-to-moderate anxiety symptoms (e.g., studies [70,71,72] and [78,79,80]), while others included participants with severe or extremely severe anxiety at baseline (e.g., studies [75,77]). It appears that individuals with more severe initial anxiety may experience more pronounced symptom reductions with tDCS, suggesting that baseline severity could moderate treatment response.

Electrode placement and montage were also important factors. While most studies targeted the DLPFC, the specifics of which hemisphere received the anode or cathode differed among trials. Some employed anodal stimulation over the right DLPFC and cathodal stimulation over the left (e.g., studies [75,76,77,78]), while others reversed this setup or placed the electrodes bilaterally. Although no single configuration emerged as definitively superior, several successful trials (e.g., study [77], which reported large reductions in anxiety) utilized right-anodal/left-cathodal stimulation. This configuration aligns with neurocognitive models suggesting a key role for the right DLPFC in emotion regulation [93]. Future research should systematically compare electrode montages to identify configurations that best modulate anxiety-related circuits in SUD populations.

Furthermore, treatment duration and frequency varied significantly across studies. Some applied relatively brief interventions (e.g., five sessions over one week), while others administered multiple sessions over several weeks. Longer courses of treatment may yield more enduring anxiety reductions, although this remains speculative given the heterogeneity of protocols. Another important consideration is whether combining tDCS with other therapeutic modalities can enhance anxiolytic effects. Studies that introduced concurrent interventions, such as alcohol cue inhibitory control training (AICT) in study [73] or Mindfulness-Based Substance Abuse Treatment (MBSAT) in study [81], provide intriguing insights. While study [73] primarily focused on depression and relapse without demonstrating a pronounced synergy for anxiety, study [81] documented stronger anxiolytic outcomes when tDCS was combined with mindfulness training compared to either intervention alone. This suggests that tDCS may optimize the neural environment for learning new coping strategies, enhancing the effectiveness of psychosocial interventions in reducing anxiety.

It is also worth noting that several studies reported improvements that approached significance or trended in a favorable direction. This pattern may indicate subtle but meaningful physiological shifts that could achieve statistical robustness with larger sample sizes or more targeted protocols. In some cases, sham groups also exhibited improvements, potentially due to non-specific factors such as therapeutic contact, expectancy effects, or concurrent standard treatments like methadone maintenance therapy. These non-specific improvements underscore the importance of rigorous control conditions and larger trials to confidently attribute observed changes to the tDCS intervention itself.

Lastly, the heterogeneity in anxiety measures—ranging from the Hamilton Anxiety Rating Scale (HAM-A) to the Beck Anxiety Inventory (BAI) and the anxiety subscale of the Depression, Anxiety, and Stress Scale (DASS-21)—introduces another variable. Although all are validated instruments, they differ slightly in sensitivity and clinical thresholds, which may influence the detection of between-group differences. Future research should consider employing standardized and consistent measures or analyzing multiple instruments in parallel.

Based on the presented evidence, tDCS protocols that apply anodal stimulation over the right DLPFC (F4) and cathodal stimulation over the left DLPFC (F3) appear to be the most consistently effective in reducing anxiety among individuals with substance use disorders. Studies [75,76,78,79], which adopted this montage, reported notable improvements in anxiety outcomes compared to sham stimulation or alternate configurations. For instance, study [75] showed a statistically significant reduction in anxiety favoring active tDCS, while studies [76,78,79] demonstrated meaningful improvements with the anode placed over the right DLPFC. Although study [77] suggested that both right-anode/left-cathode and left-anode/right-cathode setups could substantially decrease anxiety, the broader pattern across multiple investigations supports the right-anodal configuration as more consistently beneficial. Contributions from studies [72] and [81], although not always individually reaching formal significance, reinforce the trend that right-anodal tDCS can yield positive anxiety outcomes. Taken together, these findings indicate that the most effective tDCS protocol for improving anxiety scores in addiction populations involves placing the anode over the right DLPFC and the cathode over the left DLPFC.

From a clinical standpoint, these findings suggest that tDCS-driven mood improvements may act as a critical catalyst in the broader recovery process by simultaneously easing negative affect and attenuating the cognitive biases that perpetuate substance use. Effective modulation of the DLPFC could not only alleviate depressive and anxious symptoms but also positively influence executive functions—such as impulse control and decision-making—which are often impaired in SUD populations. This, in turn, may help disrupt the cyclical interplay between adverse mood states and substance-seeking behaviors. Notably, several studies point to the possible added value of pairing tDCS with behavioral or psychotherapeutic interventions, hinting that improved mood and enhanced neural plasticity can optimize patients’ receptivity to therapeutic strategies that foster long-term abstinence. Such synergy highlights the potential of tDCS as an adjunctive tool to enhance overall treatment engagement and sustain meaningful recovery outcomes in substance-using individuals.

In summary, while the evidence remains mixed, there is an emerging trend suggesting that tDCS targeting the right DLPFC can alleviate anxiety symptoms in individuals with SUD. The most robust findings come from studies involving severe baseline anxiety and active tDCS interventions that yield notable between-group differences. Factors such as baseline symptom severity, electrode montage, session frequency, and the integration of complementary therapies appear to influence outcomes. Further large-scale and well-controlled studies are needed to refine stimulation protocols, identify patient subgroups most likely to benefit from them, and integrate tDCS with other treatment modalities to achieve sustained reductions in anxiety among substance-abusing populations.

In addition to reporting raw score changes, our analysis further quantified improvements as percentage reductions in symptom measures. Across studies, active tDCS interventions yielded reductions in depression and anxiety scores ranging from approximately 20% to nearly 50%, with such improvements often corresponding to shifts from more severe to milder symptom categories. It is important to note that most studies employed relatively brief treatment courses (typically 5–10 sessions) and assessed outcomes immediately post-intervention, leaving the long-term durability of these effects largely unexamined. Therefore, while the observed percentage changes suggest a potentially clinically meaningful impact in the short term, future research should evaluate whether these improvements are sustained over extended follow-up periods. Such data would help clarify the minimal clinically important differences needed to achieve lasting functional recovery in individuals with substance use disorders.

## 5. Potential Mechanisms of Action of tDCS in Alleviating Depressive and Anxiety Symptoms in SUD

**Figure 2:**
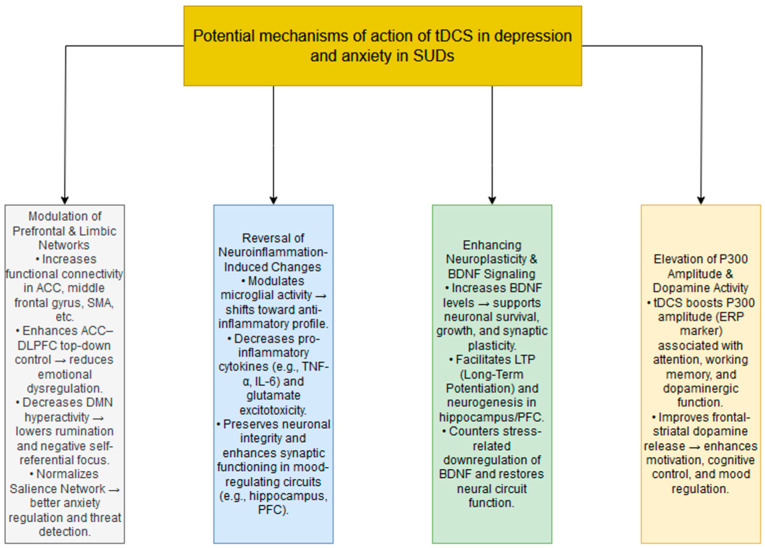
Flowchart describing potential mechanisms of action of tDCS in depressive and anxiety symptoms in substance use disorders.

The therapeutic effects of tDCS in reducing symptoms of depression and anxiety in SUD are complex and not yet fully understood. Discussing the potential mechanisms of tDCS action may facilitate future research in this area. It should be emphasized, however, that these hypothetical mechanisms are speculative in nature and require further validation through studies involving patients diagnosed with depression and anxiety in the context of SUD. A graphical representation of the mechanisms of tDCS action is presented in Figure 2.

### 5.1. Modulation of Networks by tDCS

In study [94], prefrontal tDCS increased global brain network efficiency and decreased global clustering in patients with alcohol use disorder, indicating improved integration of brain networks. Specific prefrontal sub-networks showed improved connectivity, particularly within the right anterior cingulate cortex (ACC), middle frontal gyrus, and supplementary motor area. Evidence from study [95] showed that reduced connectivity of the ACC with the DLPFC is associated with depressive symptoms. Specifically, greater depressive symptom severity was linked to decreased functional connectivity between the subgenual ACC (sACC) and DLPFC, as well as other regions of the default mode network (DMN), such as the middle frontal gyrus.

The ACC, particularly the subgenual ACC, is crucial for emotion regulation [96] and reward processing [97]. Reduced connectivity between the ACC and the DLPFC impairs the brain’s ability to regulate negative emotions and suppress maladaptive thoughts, contributing to rumination and persistent negative mood seen in depression. The ACC acts as a bridge between emotional (limbic) and cognitive (prefrontal) systems [98], and dysfunctional connectivity weakens the top-down control from the DLPFC over limbic areas, exacerbating emotional dysregulation and anxiety-like symptoms. Disrupted ACC–DLPFC connectivity may hinder cognitive control processes necessary for suppressing negative thoughts and regulating mood.

The findings from study [94] show that tDCS enhances connectivity within the prefrontal cortex, including the ACC and middle frontal gyrus. These changes counteract the reduced connectivity typically observed in depressed patients. Enhanced ACC–DLPFC connectivity likely restores top-down emotional regulation, improving control over limbic hyperactivity and reducing the emotional and cognitive symptoms of depression and anxiety. Furthermore, regions such as the middle frontal gyrus and supplementary motor area (SMA) play key roles in behavioral regulation [99], motor planning [100], and the suppression of automatic responses [101]. Dysfunction in these areas is often observed in depression and anxiety [102,103]. Improved connectivity, as reported in study [94], facilitates behavioral control and goal-directed actions, which are commonly impaired in mood disorders [104,105,106,107].

The reduced connectivity between the ACC and DLPFC aligns with impaired emotional and cognitive control mechanisms in depression and anxiety [108,109,110]. By improving functional connectivity within prefrontal sub-networks, particularly between the DLPFC, ACC, middle frontal gyrus, and SMA, tDCS addresses the underlying dysfunction. This enhanced connectivity likely strengthens cognitive control, reduces emotional dysregulation, and mitigates symptoms of depression and anxiety in individuals with SUD, where similar mechanisms are disrupted.

The fMRI results of study [111], using diffusion tensor imaging (DTI), demonstrated increased connectivity (higher fractional anisotropy and apparent diffusion coefficient values) between the vmPFC and NAcc following tDCS treatment. Importantly, this increase in connectivity correlated with reductions in craving scores for crack-cocaine use. The antidepressant effects of tDCS in SUD are closely tied to its ability to restore vmPFC function. Dysfunctional connectivity between the vmPFC and NAcc is also associated with depression [112], where impairments in reward valuation and decision-making are common. By enhancing this connectivity, tDCS alleviates depressive symptoms and reinforces prefrontal-limbic circuits involved in emotional and behavioral regulation.

Study [113] demonstrated that tDCS applied over the DLPFC led to a reduction in default mode network (DMN) connectivity. The DMN is a large-scale brain network active during resting states or self-referential tasks when an individual is not engaged in externally focused cognitive activity [114]. Key regions of the DMN include the medial prefrontal cortex (mPFC) [115], posterior cingulate cortex (PCC) [116], precuneus [117], medial temporal lobes (MTLs) [118], and inferior parietal lobules [119]. The DMN is involved in introspection, self-referential processing, and mind wandering, playing a crucial role in integrating information about the self and the environment [114].

In depression, the DMN often shows increased functional connectivity and hyperactivity, particularly in its anterior components, such as the mPFC [120]. This hyperconnectivity is associated with rumination, persistent and repetitive negative thoughts focused on negative emotions and past experiences, impaired emotional regulation, and a heightened negative self-referential focus [120]. Increased DMN connectivity disrupts interactions with task-positive networks, such as the Cognitive Control Network (CCN) and the Salience Network (SN), impairing the ability to regulate emotions effectively [121]. Dysfunctional interactions perpetuate maladaptive thought patterns, reinforcing cognitive biases and negative mood. Contributing mechanisms include disrupted network interactions where the DMN becomes hyperconnected internally but loses its balance with task-positive networks, amplifying self-focused thoughts. Neurotransmitter imbalances in glutamatergic and GABAergic systems exacerbate DMN hyperactivity, leading to reduced network inhibition [122]. Impaired top-down regulation between the hyperconnectivity. The DLPFC and the DMN limit cognitive control over excessive self-referential processing [120], while structural abnormalities, such as reduced gray matter volume within the anterior DMN, further contribute to hyperconnectivity.

tDCS applied over the DLPFC reduces DMN hyperconnectivity by enhancing top-down regulation, increasing DLPFC activity, and improving its connectivity with the ACC, restoring balance between the task-positive networks (like the CCN) and the DMN, leading to decreased hyperactivity in the anterior DMN regions, such as the mPFC and PCC, which are overly active in depression [123,124]. tDCS facilitates better network integration, improving communication between the DMN and executive control networks, which enhances cognitive control over maladaptive ruminative processes. Simultaneously, study [113] found that tDCS increased connectivity within the Salience Network (SN). The SN, which includes the anterior insula (AI) and dorsal anterior cingulate cortex (dACC), is responsible for detecting and prioritizing emotionally and behaviorally relevant stimuli. It functions as a switch between the DMN, which is active during self-referential and passive states, and the frontoparietal network (FPN), which governs task execution and cognitive control. In anxiety disorders, the SN exhibits reduced connectivity, contributing to heightened sensitivity to external and internal stimuli [125]. tDCS applied over the DLPFC modulates and enhances the SN’s function, leading to improvements in anxiety symptoms through several mechanisms. tDCS can reduce AI hyperactivity and normalize AI–dACC connectivity, restoring the SN’s ability to detect and appropriately prioritize relevant stimuli. Moreover, by balancing SN activity, tDCS prevents the over-allocation of attention to non-salient anxiety-inducing stimuli, thereby reducing hypervigilance and perceived threat sensitivity.

### 5.2. tDCS Can Reverse Neuroinflammation-Induced Brain Functional Changes

Neuroinflammation involves a range of immune-related events within the central nervous system (CNS), initiated by cells such as microglia and astrocytes and characterized by the release of pro-inflammatory signals—cytokines and chemokines—and harmful reactive oxygen compounds [126]. While inflammation is generally a protective response, prolonged neuroinflammation disrupts the brain’s delicate balance, leading to neuronal damage and impairing the brain’s adaptive and reorganization capabilities [127]. Emerging evidence highlights the role of persistent neuroinflammation in the pathogenesis of depression, particularly MDD [128]. Approximately one-third of people diagnosed with MDD show signs of inflammation-related changes [129]. These changes may originate from chronic stress, dysregulated immune responses, or systemic inflammation, which can eventually set off immune processes within the CNS via the blood–brain barrier [130,131].

Chronic stress stands out as one of the major predictors of depression. It dysregulates the HPA axis, leading to a spike in stress hormones such as cortisol. Initially, cortisol may suppress inflammation, but prolonged exposure leads to glucocorticoid resistance, exacerbating immune activation [132]. Systemic inflammatory molecules, such as TNF-α and IL-6, can enter the CNS by exploiting the BBB vulnerabilities or using specific transport pathways [133]. Alternatively, inflammation signals can reach the brain indirectly via sensory nerves, including the vagus nerve, conveying the message that the body is inflamed [134]. Once these signals reach the brain, microglia ramp up the production of inflammatory agents—cytokines, chemokines, and reactive oxygen species—intensifying neuroinflammation [130]. This hostile environment disrupts the brain’s equilibrium, impairs neurogenesis, and undermines neurotransmission, all of which contribute to depressive symptoms [135,136].

Microglial activation drives much of this inflammation-related pathology in depression [137]. When activated by stressors or inflammatory signals, microglia overproduce glutamate, leading to excitotoxicity—an overexcitation of neurons that causes cellular damage [138]. In the hippocampus, a structure vital for governing mood, memory, and various cognitive tasks, this glutamate imbalance and the concurrent drop in brain-derived neurotrophic factor (BDNF) disrupt synaptic plasticity and neurogenesis [139]. Over time, such neuroinflammatory insults contribute to memory impairments, emotional dysregulation, and cognitive deficits frequently observed in depression. Imaging studies have corroborated this, revealing reduced hippocampal volumes in individuals with MDD, emphasizing the impact of inflammation on the brain structure [140,141].

Studies indicate that tDCS can reduce markers of neuroinflammation [142,143]. Chronic neuroinflammation impairs neuroplasticity by disrupting neurogenesis in the hippocampus. tDCS may counteract these effects by enhancing BDNF signaling and supporting the integrity of hippocampal neural circuits, thereby improving mood regulation and cognitive functions (as described in another section of this review). Additionally, tDCS may modulate microglial polarization, favoring an anti-inflammatory state over a pro-inflammatory one. This shift can protect neurons from excitotoxic damage caused by excessive glutamate and improve synaptic plasticity.

Study [144] has demonstrated that elevated inflammatory states alter neurotransmitter systems (notably dopamine and glutamate), reduce connectivity in frontostriatal and prefrontal circuits, and lead to symptoms such as anhedonia, reduced motivation, anxiety, and psychomotor slowing. In contrast, tDCS has been shown to enhance connectivity and strengthen control networks in substance use disorders that are typically disrupted by inflammation. While these studies do not directly measure inflammation, the observed changes induced by tDCS oppose the functional connectivity patterns associated with elevated inflammation. Thus, it can be proposed that tDCS may mitigate the neurocircuit-level consequences of neuroinflammation.

#### 5.2.1. Frontostriatal and Reward Circuits

Inflammatory cytokines reduce dopamine availability and disrupt reward-related circuits, including the connectivity between the ventral striatum (VS) and ventromedial prefrontal cortex (vmPFC), contributing to anhedonia and motivational deficits [144]. Study [111] demonstrated that tDCS increased connectivity, as measured by fractional anisotropy and apparent diffusion coefficient values, between the vmPFC and nucleus accumbens (NAcc), core regions of the reward system. This enhancement counters inflammation-related deficits in reward processing and may restore normal motivation and pleasure responses.

#### 5.2.2. Cognitive Control and Executive Networks

Chronic inflammation is associated with impaired executive function and reduced connectivity in prefrontal networks, often manifesting as psychomotor slowing and difficulty with cognitive control [144]. Study [94] reported that prefrontal sub-networks showed improved connectivity following tDCS, with enhancements observed in the ACC, middle frontal gyrus (MFG), and supplementary motor area. Study [145] found that active tDCS increased brain activation compared to sham in regions such as the MFG, anterior insula, inferior frontal gyrus, inferior parietal lobule, and precuneus while modulating connectivity between the right superior frontal gyrus and posterior parietal cortex—key regions within the frontoparietal network (FPN). These enhancements contrast with the disrupted executive and motor circuitry often seen under inflammatory conditions.

#### 5.2.3. Salience, Anxiety, and Emotional Processing Networks

Increased inflammation correlates with heightened activity in the amygdala, dorsal anterior cingulate cortex (dACC), and insula—structures involved in threat detection, anxiety, and emotional dysregulation [144]. Inflammatory states also reduce connectivity between these regions and prefrontal areas necessary for emotional regulation [144]. Studies [94,145] collectively indicate that tDCS can enhance connectivity and activation in prefrontal regions such as the ACC and middle frontal areas, thereby improving top-down modulation of limbic structures (e.g., amygdala and insula). Study [146] found that tDCS enhanced connectivity between the orbitofrontal cortex (OFC) and DLPFC, potentially improving the balance between reward evaluation and cognitive control—mechanisms that can counteract the emotional and motivational imbalances induced by inflammation.

#### 5.2.4. Default Mode Network (DMN) and Ruminative Processes

Chronic low-grade inflammation is linked to altered DMN connectivity, potentially reinforcing rumination, negative self-referential thought, and depressive symptoms [144]. Study [147] showed that tDCS reduced task-based connectivity within the DMN and between the DMN and ventral attention network (VAN), helping to counteract DMN hyperactivity associated with inflammation. Study [148] reported that tDCS suppressed precuneus activation (a DMN hub) and improved connectivity with task-relevant networks. By reducing DMN dominance, tDCS may alleviate ruminative processes exacerbated by inflammation.

### 5.3. Enhancing Neuroplasticity

Brain-derived neurotrophic factor (BDNF) is a member of the neurotrophin family of growth factors, essential for the survival, development, and functioning of neurons. Encoded by the *BDNF* gene located on chromosome 11 in humans, *BDNF* is synthesized as proBDNF, which is cleaved into its mature form, mBDNF [149]. BDNF primarily exerts its effects by binding to its high-affinity receptor TrkB (tropomyosin receptor kinase B), triggering downstream signaling cascades, including the MAPK (mitogen-activated protein kinase), PI3K/Akt, and PLCγ pathways. These pathways regulate neuronal survival, differentiation, synaptic plasticity (strengthening of synaptic connections), neurogenesis (generation of new neurons), and long-term potentiation (LTP), which is critical for learning and memory [150,151].

BDNF is abundantly expressed in the central nervous system, with particularly high levels in the hippocampus, prefrontal cortex, amygdala, and hypothalamus—brain regions essential for mood regulation, cognition, and memory processing [152,153]. In addition to its presence in the brain, BDNF is also detected in peripheral tissues, including blood, where its levels reflect brain function due to its ability to cross the blood–brain barrier [154].

The neurotrophic hypothesis of depression posits that reduced levels of BDNF significantly contribute to the pathophysiology of major depressive disorder [154]. BDNF is essential for maintaining neural plasticity, neurogenesis, and the structural integrity of brain regions involved in mood regulation, such as the hippocampus, prefrontal cortex (PFC), and amygdala. Clinical and preclinical studies have consistently shown that individuals with depression exhibit significantly reduced BDNF levels in the hippocampus, PFC, and peripheral blood, including plasma and serum [153]. Postmortem studies of depressed and suicidal individuals have reported decreased *BDNF* mRNA and protein expression in these brain regions [155]. Chronic stress, a major precipitant of depression, further downregulates *BDNF* expression and signaling, particularly in the hippocampus [154], leading to neuronal atrophy, impaired synaptic plasticity, and decreased neurogenesis—hallmarks of depression [150]. Antidepressant treatments, including selective serotonin reuptake inhibitors (SSRIs), electroconvulsive therapy (ECT), and physical activity, have been shown to upregulate *BDNF* expression. Increased BDNF levels after treatment often correlate with symptom improvement and recovery of neural plasticity, with treatment responders showing significantly higher BDNF levels compared to non-responders [153].

When applied to the prefrontal cortex, tDCS has been shown to increase BDNF levels in depressed and SUD patients [77] through several interconnected mechanisms.

tDCS enhances neuronal excitability by depolarizing resting membrane potentials, thereby increasing synaptic activity. This promotes activity-dependent BDNF release as BDNF secretion is tightly regulated by neural activity. Increased synaptic firing activates signaling pathways that stimulate the synthesis and release of mature BDNF (mBDNF), contributing to improved synaptic plasticity.

Prefrontal tDCS enhances signaling pathways involved in neuroplasticity, such as the *BDNF*-TrkB signaling cascade. The interaction of BDNF with its receptor, TrkB, activates downstream pathways such as MAPK/ERK, PI3K/Akt, and PLCγ, leading to increased neuronal survival, dendritic growth, and synaptic strengthening. This cascade facilitates long-term potentiation (LTP), which is critical for mood regulation and cognitive function [151].

Chronic inflammation suppresses *BDNF* expression in depressed individuals [149]. tDCS exerts anti-inflammatory effects by reducing levels of pro-inflammatory cytokines and modulating the activity of microglia. By alleviating neuroinflammation, tDCS removes inhibitory effects on *BDNF* transcription, enabling increased production and release of BDNF.

### 5.4. Increase in P300 Amplitude

Study [156] demonstrated that tDCS applied to the left DLPFC in alcoholics increased the amplitude of P300 event-related potentials (ERPs). A meta-analysis has shown that patients with depression exhibit reduced P300 amplitude [157]. The P300 component is closely linked to dopamine activity, particularly in its role in cognitive processes such as attention and working memory [158].

The P3a subcomponent of P300 is associated with frontal lobe functions, particularly attention and novelty detection, and is believed to be mediated by dopamine due to its involvement in frontal attention mechanisms. This is supported by findings that Parkinson’s disease patients, who experience dopamine deficits, exhibit reduced P300 amplitudes. Additionally, dopamine antagonists like sulpiride modulate P300 amplitude, increasing it in low-amplitude individuals and decreasing it in high-amplitude individuals [158]. The dual-transmitter hypothesis proposes that dopaminergic systems in the frontal lobe are responsible for P3a generation, while norepinephrine systems in the parietal lobe contribute to P3b generation. This dual role highlights dopamine’s influence on early attentional processes (P3a) and norepinephrine’s role in target detection and memory updating (P3b) [158].

The reduced P300 observed in conditions involving dopamine dysfunction, such as depression, underscores its role as a marker for impaired cognitive processing and attention allocation. Dopamine, a key regulator of working memory and executive function, exhibits dysfunction in depression, as evidenced by abnormalities in the P300 waveform [158,159,160,161,162].

The ability of tDCS to modulate P300 amplitude and elevate it may positively affect the dopaminergic system, alleviating depressive symptoms. This aligns with the results of studies reporting that tDCS applied prefrontally increased dopamine production in the fronto-striatal pathway [163,164,165].

## 6. Limitations and Future Directions

While the current review highlights promising findings regarding the alleviation of depressive and anxiety symptoms in patients with SUD, it also underscores several methodological limitations. Identifying these limitations and proposing solutions will aid the refinement and advancement of tDCS research.

### 6.1. Heterogeneity of Study Designs

The reviewed studies vary in tDCS parameters (electrode placement, current intensity, and session duration and frequency), participant demographics, and concurrent treatments. This heterogeneity complicates comparisons and the identification of optimal protocols. Researchers should strive to standardize protocols, addressing sources of variability systematically. For instance, adopting consistent electrode montages (e.g., F3/F4 for DLPFC stimulation) and current intensities (commonly 2 mA) applied over a fixed number of sessions would enable clearer comparisons across trials. Additionally, explicit reporting of patient demographics, SUD severity, and concurrent treatment regimens would improve reproducibility and allow nuanced subgroup analyses (e.g., distinguishing results in opioid- vs. alcohol-dependent cohorts). Finally, implementing uniform outcome measures, or at least a core battery of validated scales, would greatly enhance the potential for large-scale meta-analyses that can identify which specific tDCS parameters—and which patient profiles—are most conducive to meaningful clinical gains.

### 6.2. Small Sample Sizes

The majority of the reviewed studies recruited relatively small cohorts (often fewer than 30 per group), limiting statistical power and increasing the risk of Type I and Type II errors. This may lead to trend-level findings, suggesting the potential efficacy of tDCS but falling short of statistical significance. Conversely, small samples may amplify the impact of confounding variables (e.g., participant motivation, concurrent medication use, or severity of comorbid psychiatric conditions), leading to spurious or exaggerated conclusions. The absence of robust statistical power also makes it difficult to tease apart moderators of treatment response, such as baseline depression severity, substance type (alcohol vs. stimulants), or the presence of comorbid anxiety.

Addressing this limitation requires multi-center large-scale randomized controlled trials (RCTs). Pooling resources and recruiting participants from multiple sites can generate more representative samples, better capture heterogeneity in patient characteristics, and allow for adequately powered subgroup analyses (e.g., stratifying participants by depression severity or SUD subtype). Additionally, employing interim analyses and adaptive trial designs—where sample size calculations can be adjusted as data accumulate—can help ensure that potentially beneficial tDCS protocols are neither prematurely dismissed nor overestimated. Ultimately, larger sample sizes will yield more definitive evidence on the therapeutic impact of tDCS and clarify which specific patient subgroups are most likely to benefit.

### 6.3. Short-Term Follow-Up

Most tDCS studies in SUD populations employ relatively brief follow-up periods, often limited to immediate post-intervention assessments or a few weeks thereafter. These short-term windows fail to capture the chronic relapsing nature of SUD, where symptoms and behaviors may fluctuate substantially over months or years. Consequently, it remains unclear whether observed benefits—such as reduced anxiety or depression—persist, diminish, or evolve over time, especially in the face of repeated abstinence challenges.

Short follow-up periods also make it difficult to assess the potential cumulative or synergistic effects of ongoing booster tDCS sessions or concurrent psychosocial interventions. To address this limitation, future studies should incorporate extended longitudinal monitoring, with clinical outcomes tracked for at least 6 to 12 months post-intervention. Such longer follow-up intervals would better reflect real-world conditions in which individuals are regularly exposed to substance-related cues and stressors. Investigators might also consider administering booster tDCS sessions at set intervals (e.g., monthly) to determine whether they prolong or enhance initial gains. Including repeated assessments of relapse rates, mood symptom recurrence, and quality-of-life measures would provide a comprehensive picture of long-term efficacy. By evaluating the trajectory of symptom relief over a more extended period, researchers could clarify whether tDCS offers enduring benefits and under what circumstances adjunct treatments or periodic “maintenance” sessions might be beneficial.

### 6.4. Placebo and Expectancy Effect

Several studies noted comparable improvements in sham and active tDCS groups, potentially reflecting non-specific benefits such as therapeutic contact or participant expectations. To better isolate the genuine effects of tDCS from placebo responses, future protocols should implement enhanced blinding procedures and systematically measure participant expectancy levels. Moreover, including active comparator arms (e.g., different stimulation sites or intensities) could help disentangle placebo-related gains from true neuromodulatory effects.

### 6.5. Inconsistent Outcome Measures

A significant challenge in synthesizing findings on the impact of tDCS in SUD populations is the variability in psychometric tools used to assess depression and anxiety. While validated scales such as the Hamilton Depression Rating Scale (HAM-D), Beck Depression Inventory (BDI-II), Hamilton Anxiety Rating Scale (HAM-A), Beck Anxiety Inventory (BAI), and Depression, Anxiety, and Stress Scale (DASS-21) are frequently employed, these instruments differ in scoring ranges, diagnostic cutoffs, and sensitivity to specific symptom clusters. This heterogeneity complicates comparisons of effect sizes, clinical significance, and outcomes across studies. Additionally, inconsistent timing of outcome measurements (e.g., immediately post-intervention vs. several weeks later) further complicates efforts to assess the sustained impact of tDCS on mood symptoms. To address these issues, researchers should adopt standardized outcome measures whenever possible. A core battery of validated and widely recognized instruments—e.g., a primary clinician-rated scale and a widely used self-report measure—would facilitate consistent data collection across trials. Using multiple assessments (e.g., combining the HAM-D with the BDI-II) for each domain could help capture the multifaceted nature of mood symptoms. Investigators should also strive to harmonize follow-up intervals (e.g., baseline, immediate post-treatment, 1-month follow-up, and 6-month follow-up) so that outcomes can be compared across time in a uniform manner. By implementing these principles, future research can facilitate larger-scale data pooling, improve comparability of tDCS protocols, and clarify the extent to which symptom improvements are truly attributable to the intervention.

### 6.6. Preliminary Mechanistic Insights

Although the reviewed studies suggest that tDCS may exert clinically beneficial effects by modifying neurocircuitry (e.g., DLPFC–ACC connectivity, default mode network [DMN] hyperactivity), facilitating neuroplasticity (e.g., boosting BDNF levels), and potentially reducing neuroinflammation, most evidence remains indirect. Mechanistic conclusions often rely on correlational endpoints—such as pre-post changes in functional connectivity observed in fMRI, shifts in event-related potential (ERP) amplitudes, or simple blood-based biomarkers—rather than direct real-time measures of tDCS-induced neurochemical and molecular changes. Additionally, few studies directly link these proposed mechanisms (e.g., modulation of dopamine or glutamate systems) to objectively verified behavioral or clinical outcomes.

Without longitudinal multi-modal datasets (e.g., neuroimaging, electrophysiological recordings, and neuroimmune markers) collected at multiple time points, the precise chain of causation—whereby tDCS transitions from acute neuronal depolarization to lasting therapeutic improvements—remains largely speculative. To clarify how tDCS ameliorates depression and anxiety in SUD populations, future research should focus on methodologically rigorous biomarker-driven trials. Advanced imaging techniques (e.g., real-time fMRI and PET) applied before, during, and after stimulation could capture acute changes in neurotransmitter release or shifts in functional connectivity. Parallel measurement of inflammatory markers (e.g., IL-6 and TNF-α) and neurotrophic factors (e.g., BDNF) would help confirm whether tDCS indeed counteracts neuroinflammatory states and enhances neuroplasticity. Additionally, longitudinal designs linking biomarker data to clinically meaningful outcomes (e.g., relapse rates and sustained symptom remission) are crucial to distinguishing transient neuronal excitability changes from truly therapeutic neuroadaptations. By integrating sophisticated neurobiological assessments with robust behavioral measures, future studies can more conclusively map the mechanistic pathways through which tDCS exerts its antidepressant and anxiolytic effects in SUD populations.

### 6.7. Gender and Demographic Differences

A significant number of tDCS studies in SUD populations have predominantly included male participants, limiting the generalizability of findings to female populations. Women with SUDs may have different neurobiological and psychological responses to both addiction and tDCS due to hormonal differences, distinct addiction patterns, and higher rates of comorbid psychiatric conditions such as anxiety and PTSD [166,167,168]. Additionally, socioeconomic factors, including education level, employment status, and access to healthcare, can influence treatment adherence and outcomes [167]. Cultural differences in attitudes toward addiction treatment and mental healthcare may also affect participant engagement and response to neuromodulation therapies. The efficacy of tDCS may also differ between younger and older individuals with SUDs. Adolescents and young adults may experience different neural plasticity responses to tDCS compared to older individuals with prolonged substance use histories. Age-related cognitive decline or neuroadaptive changes due to chronic substance use may moderate the effectiveness of tDCS. To address these demographic factors, future research should aim for gender-balanced participant recruitment to determine whether tDCS effects differ based on sex, include demographic assessments to explore how socioeconomic status and cultural background influence treatment outcomes, and assess whether tDCS effectiveness varies across different age groups while considering developmental factors in younger populations. Additionally, research should explore whether tDCS protocols should be adjusted based on gender, age, and cultural background to optimize treatment efficacy. Addressing these demographic considerations can enhance the applicability of tDCS for a more diverse range of individuals with SUD-related depression.

## 7. Conclusions

In summary, this mechanistic review highlights tDCS as a promising noninvasive intervention for mitigating depression and anxiety symptoms in individuals with substance use disorders. In the case of depression, stimulation of the left DLPFC is an effective tool. In the case of anxiety, stimulation of the right DLPFC is most effective. While the studies reviewed generally indicate positive outcomes, significant questions persist regarding optimal electrode configurations, treatment duration, and the long-term sustainability of clinical improvements.

Preliminary mechanistic evidence suggests that tDCS may exert its therapeutic effects by modulating neurocircuitry (e.g., DLPFC–ACC connectivity and default mode network overactivity), enhancing neuroplasticity (e.g., through BDNF signaling), and potentially mitigating neuroinflammatory processes. However, these insights remain largely indirect, pointing to the need for more rigorous biomarker-focused research.

Future studies should further investigate the efficacy of tDCS in treating neuropsychiatric disorders in SUDs. In addition to exploring its underlying mechanisms, researchers should ensure sample homogeneity, adequate sample sizes, extended follow-up periods, effective control for placebo effects, and the use of validated measurement methods.

## Figures and Tables

**Table 1:** Summary of main findings from articles included in the review.

Key Anxiety Findings	Key Depression Findings	Anxiety Measure and Baseline	Depression Measure and Baseline	tDCS Parameters	Population and Study Design	Study (Ref.)
Active: Median change in HAM-A = −5.5 vs. Sham: −2 *p =* 0.169 => not significant	Active: Median change in HAM-D = −6 (range: −20 to −3) Sham: −1 (range: −3 to 14) *p* = 0.005 => significant reduction in active vs. sham	HAM-A Median baseline: 4 (range: 0–24)	HAM-D Median baseline: 6 (range: 0–31)	2 mA, 20 min/session Anode over F3 (left DLPFC) Cathode over contralateral supradeltoid	Alcohol dependence (*N =* 13) RCT with 2 arms: Active (*n* = 6) vs. Sham (*n* = 7) 5 weekly sessions	[70]
Post-treatment anxiety ~7.1 ± 7.3 (no anxiety) in real group; difference vs. sham was not significant	Post-treatment HAM-D ~6.8 ± 1.7 (no depression) in real group; difference vs. sham was not significant Within-group: sham improved significantly (*p =* 0.01); change in real group was not significant (*p* = 0.22)	HAM-A Real group = 11.0 ± 9.0 at baseline (mild)	HAM-D Real baseline = 8.6 ± 8.9 (mild)	2 mA, 13 min/session, twice daily Cathode over left DLPFC and anode over right DLPFC	Severe alcohol dependence (*N* = 33) RCT with 2 arms: Real tDCS (*n* = 16) vs. Sham tDCS (*n* = 17) 5 consecutive days, 2 sessions/day	[71]
HAM-A also decreased over time (*p* < 0.001). Active: 13.3 → 7.1 and Sham: 9.9 → 7.8, *p =* 0.053 => not significant	HAM-D decreased significantly over time in both groups (*p* < 0.001). Active: 14.8 → 7.6 and Sham: 12.4 → 9.1. Between-group *p* = 0.063 => not significant	HAM-A	HAM-D Baseline in Active = 14.8 ± 7.8 (mild)	1.5 mA, 20 min/day Anode over F4 (right DLPFC) and cathode over F3 (left DLPFC)	SUDs/Gambling (*N* = 34) RCT with 2 arms: Active (*n* = 18) vs. Sham (*n* = 16) 5 consecutive days	[72]
Not measured	All groups showed reductions in BDI-II: Real + AICT = 19.92 → 14.72, Real + NICT = 21.82 → 17.5, Sham + AICT = 20.52 → 15.13, and Sham + NICT = 19.26 → 16.04. No significant effect of real vs. sham or AICT vs. NICT	Not measured	BDI-II Real + AICT = 19.92 Real + NICT = 21.82 Sham + AICT = 20.52 Sham + NICT = 19.26 (All mild–moderate range)	2 mA, 20 min/day Anode over right DLPFC (F4) and cathode over left DLPFC (F3)	Severe AUD (N = 125) Randomized 2 × 2 factorial: Real vs. Sham × AICT vs. NICT 5 consecutive days with concurrent inhibitory control training	[73]
Not measured	Post: Real = 15.40 ± 4.49 (mild) => 33.16% reduction (significant, *p* < 0.0001) vs. Sham = 19.76 ± 3.80 => 2.76% reduction (*p* = 0.110)	Not measured	HDRS Baseline Real = 23.04 ± 4.44 (moderate–severe)	2 mA, 20 min/session Electrodes over F3 and F4 (DLPFC)	Cannabis use disorder (*N* = 50 males) RCT with 2 arms: Real (*n* = 25) vs. Sham (*n* = 25) 20 sessions over 10 weeks (twice/week)	[74]
Post: Real = 16.86 ± 4.20 vs. Sham = 23.23 ± 7.68 *p* = 0.01 => significantly lower anxiety in real group	Post: Real = 17.57 ± 4.45 vs. Sham = 25.85 ± 7.09 *p* < 0.001 => significant improvement in real tDCS group	DASS−21 (Anx) Real = 27.00 ± 8.25 (severe) and Sham = 25.38 ± 9.36 (severe)	DASS−21 (Dep) Real = 30.71 ± 7.75 (ext. severe and Sham = 27.85 ± 8.46 (severe)	2 mA, 20 min/session Anode on right DLPFC (F4) and cathode on left DLPFC (F3)	Opioid dependence (*N =* 27) RCT with 2 arms: Real (*n =* 14) vs. Sham (*n* = 13) 7 sessions over 2 weeks	[75]
Active = 9.7 ± 3.58 vs. Sham = 14.54 ± 4.64 vs. MMT = 14.05 ± 4.67 => *p =* 0.02 within-group for active only; sham and MMT-only not significant	Post: Active = 14.05 ± 6.18 vs. Sham = 18.30 ± 6.89 vs. MMT = 18.10 ± 6.75 *p* = 0.009 group × time, *p* = 0.01 within-group => significant improvement only in active group	BAI Active = 12.10 ± 4.47 (mild), Sham = 14.20 ± 4.76, and MMT = 14.85 ± 4.99	BDI-II Active = 17.55 ± 6.45 (mild), Sham = 18.70 ± 6.48, and MMT = 17.40 ± 7.07	2 mA, 20 min/day Anode on right DLPFC (F4) and cathode on left DLPFC (F3)	Opium use disorder (*N* = 60) 3 groups: Active tDCS + MMT (*n =* 20), Sham + MMT (*n* = 20), and MMT-only (*n* = 20) 10 consecutive days	[76]
Post: A = 9.60 (normal–mild), B = 11.82 (mild), and C = 23.20 (severe) => A = 60.97%, B = 52.20%, and C = 3.33%. *p =* 0.001 A vs. C; *p =* 0.006 B vs. C.	Post: A = 14.40 (moderate, significant), B = 17.09 (moderate, significant), and C = 26.80 => A = 48.93%, B = 41.96%, and C = −2.29% (slight, non-significant). *p =* 0.023 A vs. C; B vs. C not significant	DASS-21 (Anx) A = 24.60 ± 7.77, B = 24.73 ± 8.86, and C = 24.00 ± 7.95 (all severe)	DASS-21 (Dep) A = 28.20 ± 8.66, B = 29.45 ± 6.99 (both extremely severe), and C = 26.20 ± 7.51 (severe)	DLPFC bilaterally (2 active montages) 20 min/session; current intensity not specified	Opioid addiction (*N* = 30) 3 groups: A (anode L/cathode R), B (anode R/cathode L), and C (sham) 10 sessions	[77]
Post: Active = 6.4 vs. Sham = 8.7 => *p =* 0.03 => active tDCS effectively reduced anxiety vs. sham	Post: Active = 3.2 => *p =* 0.04 within-group (significant) and Sham = 3.5 => *p =* 0.45 between-group => not significant	HAM-A Active = 7.6 and Sham = 6.0 => no baseline difference	HAM-D Active = 5.0 and Sham = 4.3 => no baseline difference	2 mA, 20 min/session Anode over right DLPFC (F4) and cathode over left DLPFC (F3)	Crack-cocaine dependence (*N =* 36 males) RCT with 2 arms: Active (*n* = 17) vs. Sham (*n* = 19) 5 sessions, every other day	[78]
Post: Real = 5.3 vs. Sham = 9.3 => group × time *p =* 0.03 => active tDCS reduced anxiety compared to sham	Post: Real = 3.3 => *p =* 0.04 within-group (significant) and Sham = 4.8 => *p =* 0.45 between groups => no significance	HAM-A Real = 7.6 ± 5.8 and Sham = 6.0 ± 4.3 => no baseline difference	HAM-D Real = 5.6 ± 3.0 and Sham = 4.3 ± 3.1 => no baseline difference	2 mA, 20 min/session Anode over right DLPFC (F4) and cathode over left DLPFC (F3)	Cocaine use disorder (*N* = 17) RCT with 2 arms: Real (*n* = 8) vs. Sham (*n* = 9) 15 sessions over 5 weeks	[79]
No significant reduction in BAI (*p* = 0.624)	Not measured	BAI Baseline = 8.8 => minimal–mild anxiety	Not measured	2 mA, 20 min/session Anode over left DLPFC and cathode on contralateral side Beam F3 System for placement	Crack/cocaine addiction (*N* = 11 males) Open-label study, 10 consecutive daily sessions	[80]
Largest reduction in anxiety in combined group vs. single interventions or sham. All active arms significantly improved vs. sham	Greatest decrease in depression found in the combined (tDCS + MBSAT) group; both tDCS alone and MBSAT alone also > sham but not as large as the combined group	DASS-21 (Anx) Baseline ~13 (mild) from graph; no exact numerical data	DASS-21 (Dep) Baseline ~13 (mild) from graph; no exact numerical data	1.5 mA, 20 min/session Anode over left DLPFC (cathodal placement not reported)	Methamphetamine dependence (*N* = 80 adolescents) 4 groups: tDCS only, MBSAT only, Combined (tDCS + MBSAT), and Sham 12 sessions over 6 weeks	[81]

Abbreviations: AICT = Alcohol Cue Inhibitory Control Training, AUD = Alcohol Use Disorder, BAI = Beck Anxiety Inventory, BDI-II = Beck Depression Inventory-II, DASS-21 = Depression, Anxiety, and Stress Scale (21-item), DLPFC = Dorsolateral Prefrontal Cortex, F3/F4 = Standard 10–20 EEG coordinates for Left/Right DLPFC, Go/No-Go = A Response Inhibition Paradigm, HAM-A = Hamilton Anxiety Rating Scale, HAM-D/HDRS = Hamilton Depression Rating Scale, MBSAT = Mindfulness-Based Substance Abuse Treatment, MMT = Methadone Maintenance Treatment, NICT = Neutral Inhibitory Control Training, RCT = Randomized Controlled Trial, SD = Standard Deviation, and SUD = Substance Use Disorder.

## Data Availability

No new data were created or analyzed in this study. Data sharing is not applicable to this article.
